# SWI/SNF Antagonism of PRC2 Mediates Estrogen-Induced Progesterone Receptor Expression

**DOI:** 10.3390/cells11061000

**Published:** 2022-03-15

**Authors:** Mike R. Wilson, Jake J. Reske, Julie Koeman, Marie Adams, Niraj R. Joshi, Asgerally T. Fazleabas, Ronald L. Chandler

**Affiliations:** 1 Department of Obstetrics, Gynecology and Reproductive Biology, College of Human Medicine, Michigan State University, Grand Rapids, MI 49503, USA; wils1405@msu.edu (M.R.W.); reskejak@msu.edu (J.J.R.); joshini@msu.edu (N.R.J.); fazleaba@msu.edu (A.T.F.); 2 Genomics Core Facility, Van Andel Research Institute, Grand Rapids, MI 49503, USA; julie.koeman@vai.org (J.K.); marie.adams@vai.org (M.A.); 3 Department of Women’s Health, Spectrum Health System, Grand Rapids, MI 49341, USA; 4 Center for Epigenetics, Van Andel Research Institute, Grand Rapids, MI 49503, USA

**Keywords:** endometrial cancer, ARID1A, progesterone receptor

## Abstract

Endometrial cancer (EC) is characterized by high estrogen levels unopposed by progesterone. Treatment with progestins is standard for early EC, but the response to progestins is dependent on progesterone receptor (PGR) expression. Here, we show that the expression of PGR in endometrial epithelial cells is dependent on ARID1A, a DNA-binding subunit of the SWI/SNF chromatin-remodeling complex that is commonly mutated in EC. In endometrial epithelial cells with estrogen receptor overexpression, we find that ARID1A promotes estrogen signaling and regulates common gene expression programs. Normally, endometrial epithelial cells expressing estrogen receptors respond to estrogen by upregulating the PGR. However, when ARID1A expression is lost, upregulation of PGR expression is significantly reduced. This phenomenon can also occur following the loss of the SWI/SNF subunit BRG1, suggesting a role for ARID1A- and BRG1-containing complexes in PGR regulation. We find that PGR is regulated by a bivalent promoter, which harbors both H3K4me3 and H3K27me3 histone tail modifications. H3K27me3 is deposited by EZH2, and inhibition of EZH2 in the context of ARID1A loss results in restoration of estrogen-induced PGR expression. Our results suggest a role for ARID1A deficiency in the loss of PGR in late-stage EC and a therapeutic utility for EZH2 inhibitors in this disease.

## 1. Introduction

Endometrial cancer (EC) is the most commonly diagnosed gynecologic malignancy, with an estimated 66,570 new cases diagnosed in 2021 [1]. The incidence of EC is increasing at a rate of 1% year on year [1]. Although EC is historically a disease of post-menopausal women, the incidence is also increasing in pre-menopausal women, a phenomenon attributed to increasing rates of obesity [2,3]. EC is derived from the endometrium, the cells that form the inner lining of the uterus and undergo monthly proliferation, differentiation, and shedding in response to ovarian steroid hormones [4]. Hyperproliferation of the endometrium results in endometrial hyperplasia, and complex atypical endometrial hyperplasia (CAH) is associated with increased risk for EC [5,6]. 

The functions of the endometrium are highly regulated through steroid hormone signaling. During the menstrual cycle, estrogen, through estrogen receptor α (ESR1), promotes proliferation of the endometrium, while progesterone inhibits estrogen-induced proliferation and promotes secretory differentiation through progesterone receptor (PGR) [7]. ESR1 and PGR belong to a superfamily of nuclear receptors that respond to extracellular ligands, translocate to the nucleus, bind DNA and modulate transcription [8]. Under normal conditions, PGR expression represents a critical feedback mechanism to temper the proliferative effects driven by estrogen [8]. 

In several endometrial pathologies, including EC and endometriosis, the balance between estrogen signaling and progesterone signaling becomes perturbed, either through increased reliance on estrogen or diminished responsiveness to progesterone [9,10]. In the case of EC, peripheral aromatization of androgens in the adipose tissue results in increased estrogen in obese women [2]. Most endometrial hyperplasias and 50–70% of endometrial cancers are responsive to progestin therapy, which is a current standard of care treatment for CAH and early EC [5,6,11]. Expression of PGR is an important prognostic factor in determining whether EC will respond to progestin therapy or if the tumor has become progesterone resistant [11,12]. Poor progestin response rates are correlated with disease recurrence [11]. Loss of PGR is observed in both CAH and EC [13,14]. Additionally, PGR expression is predictive of progestin response in endometriosis [15]. 

Genetic factors are associated with increased EC risk, including somatic mutations in ARID1A, a DNA-binding subunit of the SWI/SNF chromatin-remodeling complex. ARID1A is mutated in up to 16% of CAH and 40% of EC, and ARID1A mutations are more frequent in women diagnosed with EC at a younger age [16,17,18,19,20,21]. ARID1A is also associated with malignant transformation [17]. SWI/SNF chromatin remodeling alters the epigenome of cells, leading to transcriptional activation or repression of genes [22]. Previously, we have shown that SWI/SNF is an important regulator of cell identity in the endometrial epithelium, such that loss of ARID1A or the SWI/SNF catalytic subunit BRG1 can contribute to invasive endometrial pathologies [23,24,25,26,27]. This study aims to understand the role of SWI/SNF in regulating steroid hormone processes in the endometrial epithelium and the effect this may have on endometrial cancer pathogenesis. We performed transcriptome analysis of ESR1-positive endometrial epithelial cells, with and without ARID1A expression, and found that 55% of estrogen-altered genes are also affected by ARID1A loss. Among co-altered genes, we show that ARID1A is required for the estrogen-dependent activation of estrogen target genes, including *PGR*. We further show that a subset of estrogen and ARID1A co-regulated gene promoters contain histone post-translational modifications associated with bivalent chromatin. Bivalent chromatin-containing genes are repressed by the polycomb repressive complex 2 (PRC2) yet remain poised for transcriptional activation [28]. Drug inhibition of PRC2 methyltransferase subunit EZH2 partially rescues the effects of ARID1A loss on estrogen-dependent activation of PGR, supporting a role for PRC2 activity in *PGR* gene repression. Our results suggest that when ARID1A is mutated, estrogen-dependent transcription of the critical estrogen target gene, *PGR*, does not occur because of PRC2 activity at the PGR locus, resulting in progesterone resistance and increased EC cancer risk.

## 2. Methods

### 2.1. Cell Lines

12Z immortalized human endometrial epithelial cells [29] were maintained in DMEM/F12 media without phenol red (Gibco, Billings, MT, USA; cat# 11039-021) supplemented with 10% charcoal-stripped fetal bovine serum (FBS) (Gibco; cat# A33821-01), 1% L-glutamine and 1% penicillin/streptomycin (P/S). 12Z cells were transfected with the *ESR1* plasmid from the Precision LentiORF Collection (Horizon Discovery Biosciences, Cambridge, UK) and grown under Blasticidin conditions. Stable expression was confirmed by western blot.

### 2.2. Transfection and Treatment of 12Z Cells with siRNA

12Z-ESR1 cells were seeded at a density of 40,000 cells/mL DMEM/F12 media without phenol red supplemented with 10% FBS and 1% L-glutamine. The following day, the media was refreshed, and cells were transfected with 50 pmol/mL of siRNA (Dharmacon, Lafayette, CO, USA; ON-TARGETplus Non-targeting Pool and human ARID1A #8289 SMARTpool) using the RNAiMax (ThermoFisher, Waltham, MA, USA) lipofectamine reagent according to the manufacturer’s instructions at a ratio of 1:1 volume:volume in OptiMEM (Gibco, Billings, MT, USA). After 24 h, the media was replaced with DMEM/F12 media without phenol red supplemented with 10% charcoal-stripped FBS 1% L-glutamine. The following day, the media was replaced with DMEM/F12 media without phenol red supplemented with 0.5% charcoal-stripped FBS, 1% L-glutamine containing either 10 nM β-Estradiol (Sigma, St. Louis, MO, USA; cat# E2758) or 0.1% ethanol vehicle.

### 2.3. Western Blotting

Protein lysates were quantified using the Micro BCA Protein Assay Kit (ThermoFisher) and a FlexSystem3 plate reader. Protein lysates were run on a 4–15% gradient SDS-PAGE gel (BioRad, Hercules, CA, USA) and transferred to the PVDF membrane using the TransBlot Turbo system (BioRad). Primary antibodies were used at the following dilutions: 1:1000 ARID1A (D2A8U) (12354, Cell Signaling, Danvers, MA, USA); 1:1000 β-Actin (8457, Cell Signaling); 1:1000 PGR (SAB5500165, Sigma); 1:1000 ESR1 (MCA1799, Bio-Rad), 1:100 BRG1 (sc-17796, Santa Cruz Biotechnology, Dallas, TX, USA); 1:1000 ARID1B (92964, Cell Signaling). Horseradish peroxidase (HRP) conjugated secondary antibodies (Cell Signaling) were used at a dilution of 1:2000. Clarity Western ECL Substrate (BioRad) was used for protein band visualization, and western blot exposures were captured using the ChemiDoc XRS+ imaging system (BioRad). Uncropped western blots are shown in Supplementary Figure S4. 

### 2.4. RNA Isolation and qRT-PCR

RNA samples were collected 72h-post siRNA transfection using the Quick-RNA Miniprep Kit (Zymo Research, Irvine, CA, USA). cDNA was synthesized from RNA, and qRT-PCR was performed using PowerUp SYBR Green Master Mix (ThermoFisher, Waltham, MA, USA) and the Applied Biosystems (Waltham, MA, USA) ViiA7 real-time PCR system. 

### 2.5. Construction and Sequencing of Directional mRNA-seq Libraries

Libraries were prepared by the Van Andel Genomics Core from 500 ng of total RNA using the KAPA mRNA HyperPrep kit (v4.17) (Kapa Biosystems, Wilmington, MA, USA). RNA was sheared to 300–400 bp. Prior to PCR amplification, cDNA fragments were ligated to Bioo Scientific NEXTflex dual indexing adapters (Bioo Scientific, Austin, TX, USA). Quality and quantity of the finished libraries were assessed using a combination of Agilent DNA High Sensitivity chip (Agilent Technologies, Inc, Santa Clara, CA, USA.), QuantiFluor^®^ dsDNA System (Promega Corp., Madison, WI, USA), and Kapa Illumina Library Quantification qPCR assays (Kapa Biosystems, Wilmington, MA, USA). Individually indexed libraries were pooled and 75 bp, single-end sequencing was performed on a NextSeq500 sequencer using a 75-cycle high output kit (v2) (Illumina Inc., San Diego, CA, USA) and each library was sequenced to an average raw depth of 35 M reads. Base-calling was done by Illumina NextSeq Control Software (NCS) v2.0 and the output of NCS was demultiplexed and converted to FastQ format with Illumina Bcl2fastq v1.9.0.

### 2.6. RNA-seq Analysis

Raw 75 bp reads were trimmed with *cutadapt* [30] and *Trim Galore!* (http://www.bioinformatics.babraham.ac.uk/projects/trim_galore/, accessed on 11 March 2022) followed by quality control analysis via *FastQC* [31]. Trimmed reads were aligned to GRCh38.p12 and indexed to GENCODE [32] v28 annotation via *STAR* [33] aligner with the flag ‘--quantMode GeneCounts’ for feature counting. Output gene count files were constructed into an experimental read count matrix in R. Low count genes were filtered (1 count per sample on average) prior to *DESeq2* [34,35] count normalization and subsequent differential expression analysis. Calculated differential expression probabilities were corrected for multiple testing by independent hypothesis weighting (IHW) [36] for downstream analysis. Differentially expressed gene thresholds were set at FDR < 0.0001. Principal component analysis was calculated using *DESeq2* from the top 500 genes by variance across samples. Relative gene expression heatmaps were produced using relative regularized logarithm (rlog) [34] counts by subtracting mean rlog counts of the control group. Relative linear gene expression bar plots were produced from the *DESeq2* normalized counts table.

### 2.7. Analysis of PGR Chromatin from Roadmap

NIH Roadmap Epigenomics data were used to explore the relationship between H3K27me3 and PGR expression in human tissues [37]. Broad H3K27me3 domains were analyzed on the Roadmap Web Portal (https://egg2.wustl.edu/roadmap/web_portal/ accessed on 28 December 2021), and samples were binarized by presence or absence of any H3K27me3 peak calls within 3 kb of the PGR gene TSS. Among the 56 samples with matched RNA-seq data, PGR gene expression (quantified by RPKM, Reads Per Kilobase of transcript, per Million mapped reads) was then compared between samples with vs. without promoter H3K27me3. 

### 2.8. Bioinformatics and Statistics

Wild-type 12Z cell ARID1A ChIP-seq data and siARID1A vs. control 12Z cell total RNA-seq data were re-analyzed from GSE121198 as previously described [24]. Genes exhibiting bivalent promoter chromatin in 12Z cells were identified from previously reported H3K4me3 and H3K27me3 ChIP-seq data GSE148474 [25]. Active super-enhancer regions were previously defined [25] from H3K27ac ChIP-seq data which overlapped with accessibility (ATAC) [38] in control 12Z cells using the ROSE algorithm [39,40]. GeneHancer [41] database was used to associate enhancers to genes with a GeneHancer score > 1 threshold. HOMER [42] was used to count sequencing reads at genomic sites of interest and quantify signal and count reads at sites of interest for tag density heatmaps and meta peak plots. Chromatin analyses involving ChIP signal quantification at regions of interest used pooled reads from both IP replicates, per feature. TxDb.Hsapiens.UCSC.hg38.knownGene [43] was used to define gene promoters for all standard hg38 genes as 3 kilobase regions surrounding the primary TSS. MACS2 [44] was used to produce genome-wide signal log-likelihood ratio (logLR) tracks for *IGV* [45] visualization. ClusterProfiler [46] was used to compute and visualize pathway enrichment from a list of gene symbols with respective gene universes. Hallmark pathways and GO Biological Process gene sets were retrieved from MSigDB [47]. ComplexHeatmap [48] was used for hierarchical clustering by Euclidean distance and general heatmap visualization. GenomicRanges [49] functions were frequently used to intersect and manipulate genomic coordinates, for example, for genome-wide association tests. eulerr [50] was used to produce proportional Euler diagrams. biomaRt [51,52] was used for all gene nomenclature and ortholog conversions. ggplot2 [53] was used for certain plotting applications. The statistical computing language R [54] was used for many applications. ImageJ software (National Institutes of Health, Bethesda, MD, USA) was used to calculate western blot densitometry. GraphPad Prism 8 software was used to perform *t*-tests.

Endometrial cancer cell line data for 40 cell lines were collected from the Cancer Cell Line Encyclopedia (CCLE) [55] using the CCLE DepMap tool. Endometrial and breast cancer patient sample mRNA and protein data from The Cancer Genome Atlas PanCancer Atlas dataset [56] were accessed through cBioPortal [57,58].

### 2.9. Cell Growth Assay

12Z cells were seeded at a density of 4000 cells per well in a 96-well plate. After 24 h, cells were treated with EPZ-6438 at concentrations from 100 nM to 100 μM, or 0.1% DMSO vehicle. After 48 h, cells were incubated with 2 μg/mL calcein-AM for 1 h, and fluorescence was measured using a SpectraMax i3x (Molecular Devices, San Jose, CA, USA).

## 3. Results

### 3.1. ARID1A and Estrogen Signaling Cooperatively Regulate Biological Processes in the Endometrial Epithelium

**Figure 1:**
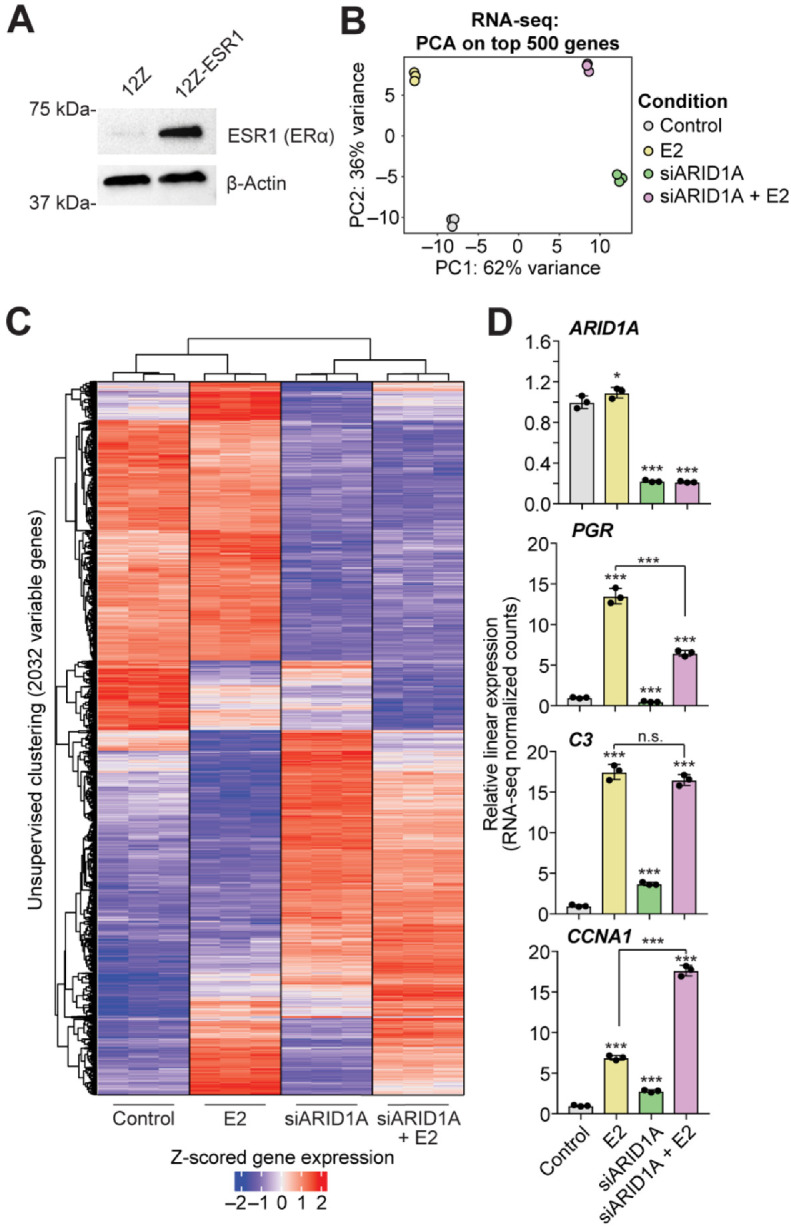
Differential gene expression response to estrogen signaling following the loss of ARID1A expression. (**A**) Confirmation of ESR1 expression in 12Z-ERS1 cells by western blot. β-Actin was used as a loading control. (**B**) Principal component analysis on top 500 variable genes from RNA-seq on 12Z-ESR1 cells following treatment with 10 nM estrogen (E2, yellow), siRNA knockdown of ARID1A (siARID1A, green) or E2 + siARID1A (pink) compared to control (silver). (**C**) Unsupervised clustering of samples based on the expression of 2032 genes (rows) selected by variance across the experimental design (rlog variance > 0.05). Heatmap displays relative expression as Z-scored rlog counts. Red values indicate high relative gene expression, and blue values indicate low expression. (**D**) Examples of gene expression alterations following treatment with E2 (yellow), siARID1A (green) or E2 + siARID1A (pink) compared to control (silver). *Y*-axis represents linear relative gene expression with respect to the control condition. Means ± SDs, *n* = 3. The statistic is *DESeq2* FDR. * *FDR* < 0.05, *** *FDR* < 0.001, n.s. is non-significant.

**Figure 2:**
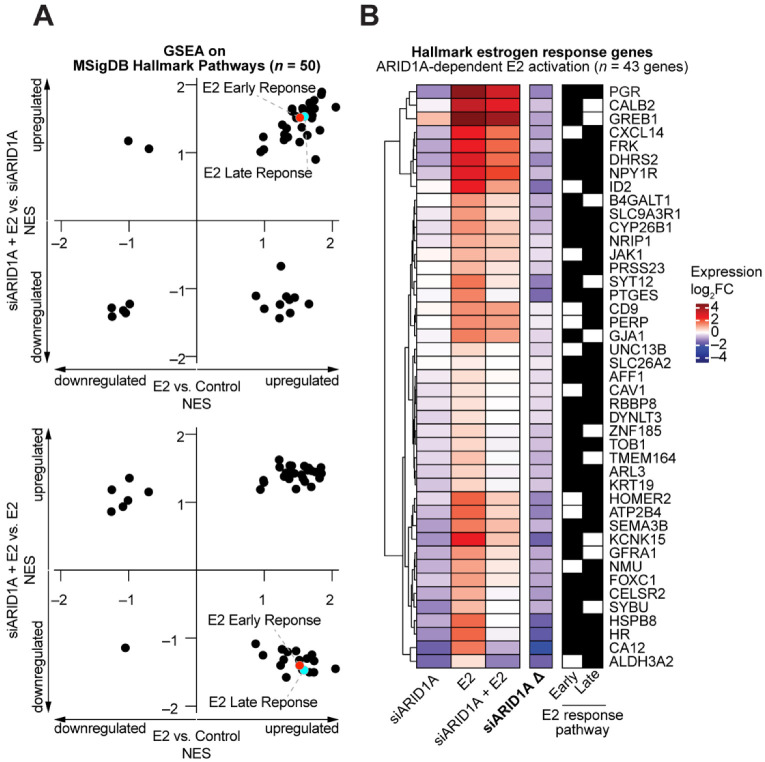
Perturbations in estrogen response following ARID1A loss. (**A**) Broad Gene Set Enrichment Analysis (GSEA) across RNA-seq conditions. Samples were tested for enrichment of the MsigDB hallmark pathways in the described comparisons, and Normalized Enrichment Scores (NES) are plotted to highlight differences between E2 vs. Control (x-axis) and siARID1A + E2 vs. siARID1A (*y*-axis, top) or siARID1A + E2 vs. E2 (y-axis, bottom). A full list of individual pathway values are provided in Table 1. E2 Early Response (red) and E2 Late Response (cyan) pathways are indicated. (**B**) 43 genes within the MsigDB Hallmark early or late estrogen response pathways that are significantly induced by E2 treatment but to a significantly less extent in the context of ARID1A knockdown. Left, clustered heatmap of expression log2FC values in siARID1A, E2, or siARID1A + E2 conditions compared to control cells. siARID1A Δ represents the change in expression between siARID1A + E2 vs. siARID1A conditions. Blue cells indicate downregulation, and red cells indicate upregulation. Right, black cells indicate membership to Hallmark estrogen response early and late pathway gene sets.

Most endometrial cancer cell lines do not express ESR1 and harbor ARID1A mutations (Figure S1) as well as other cancer-driver mutations [55]. Therefore, to investigate the role of estrogen signaling in endometrial epithelial cells, we utilized the immortalized 12Z cell line [29]. 12Z cells were transfected with the Precision LentiORF (horizon) plasmid containing a human *ESR1* open reading frame cDNA to develop a cell line with stable ESR1 expression. Stable expression of *ESR1* was developed under selection with Blasticidin. Protein expression of ESR1 was confirmed by western blot (Figure 1A). Then, 12Z cells with stable expression of *ESR1* (henceforth, 12Z-ESR1 cells) were transiently transfected with ARID1A siRNAs (siARID1A) or non-targeting RNAs [24]. After transfections, 12Z-ESR1 cells were treated with 10 nM estrogen (estradiol, E2) or vehicle for 24 h. At this time, RNAs were collected for transcriptome analysis by RNA-seq. Principal component analysis based on gene expression revealed clear segregation by ARID1A knockdown or E2 treatment (Figure 1B). Unsupervised clustering of 2032 variable genes revealed that ARID1A loss had a larger effect on gene expression than E2 (Figure 1C). However, numerous patterns of estrogen-regulated gene expression were observed, including genes that are dependent upon ARID1A for estrogen-mediated induction (*PGR*), not augmented by ARID1A loss (*C3*), or augmented by ARID1A loss (*CCNA1*) (Figure 1D). As with *PGR*, *C3* is a well-defined ESR1 target gene [59,60,61], suggesting high variability in the role of ARID1A in regulating estrogen-induced, ESR1-dependent gene expression. We performed Broad Gene Set Enrichment Analysis (GSEA) on these RNA-seq data and observed that many of the MSigDB Hallmark pathways were significantly upregulated by E2 treatment independent of ARID1A loss (Table 1). Pathways related to estrogen signaling itself (estrogen response early, estrogen response late) appeared to be upregulated upon E2 treatment but reduced in ARID1A knockdown cells treated with E2 compared to E2 treatment alone (Figure 2A). We examined the expression of each gene in these pathways and identified 43 Hallmark estrogen response genes that display ARID1A-dependent induction by estrogen (Figure 2B). These results suggest that ARID1A is required for the expression of certain estrogen target genes in response to estrogen stimulation.

**Figure 3:**
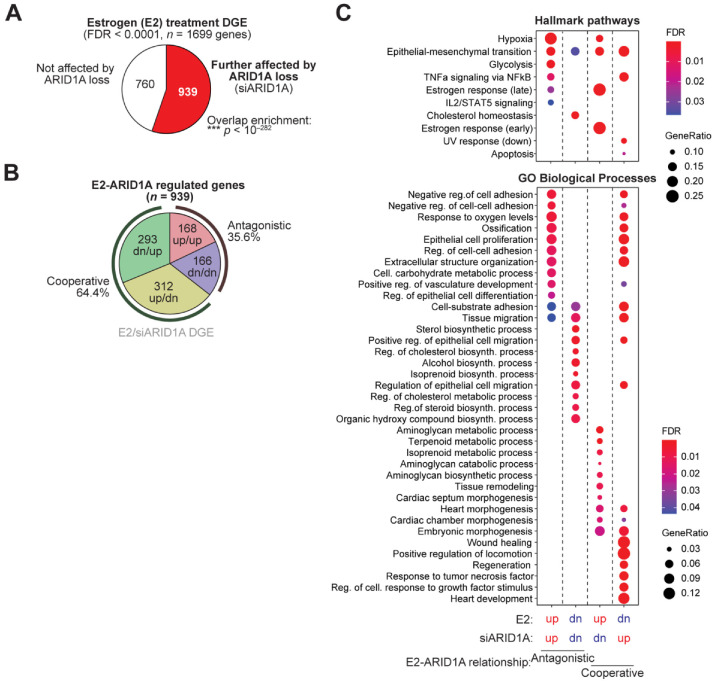
Exploring ARID1A-mediated estrogen response. (**A**) Overlap of 1699 empirical estrogen response genes and siARID1A DGE in 12Z-ESR1 cells. Those further affected by ARID1A loss (red) or not affected by ARID1A loss (white). (**B**) Directional classification of perturbed genes mutually regulated by E2 and ARID1A. Normal ARID1A regulation is inferred as the opposite effect of siARID1A treatment. Genes upregulated in both conditions (red), downregulated in both conditions (blue), upregulated by E2 and downregulated by siARID1A (yellow) or downregulated by E2 and upregulated by ARID1A (green). (**C**) Gene set enrichment analysis for MsigDB Hallmark pathways (top) and GO Biological Processes (bottom) among E2-ARID1A regulated gene classes.

Among the 1699 genes significantly differentially expressed upon E2 treatment of 12Z-ESR1 cells (FDR < 0.0001), 939 were further affected by the loss of ARID1A (Figure 3A). Most overlapping genes (64.4%) were normally cooperatively regulated by E2 and ARID1A (i.e., different direction of perturbation with ARID1A loss and E2 treatment, suggesting that normal ARID1A function is cooperative with E2), with the remaining 35.6% of genes classified as exhibiting antagonistic regulatory activity (Figure 3B). Gene set enrichment analysis was then performed for MsigDB Hallmark pathways and GO Biological Processes. Strikingly, Hallmark estrogen target genes were enriched among genes cooperatively activated by E2 and ARID1A, while EMT, cell adhesion, and wound healing responses were often cooperatively downregulated in response to estrogen (Figure 3C). Furthermore, E2 and ARID1A appear to regulate metabolic pathways, including glycolysis and cholesterol homeostasis, antagonistically (Figure 3C). These results suggest ARID1A facilitates estrogen-mediated gene expression, both among genes induced and repressed by estrogen signaling. Known pathological processes regulated by ARID1A in endometrial models [23,24,25,26], like EMT, migration, adhesion, and wound healing, are indicated as being normally suppressed by estrogen signaling, which supports cooperative roles for ARID1A and estrogen signaling in the maintenance of endometrial epithelial identity.

### 3.2. Cell Identity of the Endometrial Epithelium Depends on ARID1A Expression and Regulation by Estrogen

**Figure 4:**
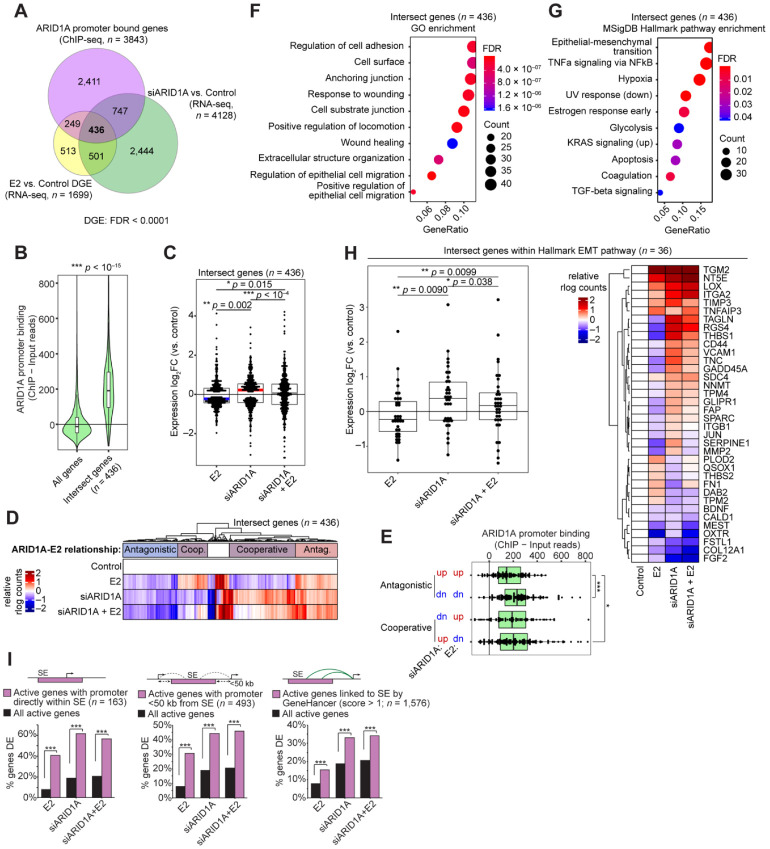
ARID1A directly regulates estrogen-induced changes to cell identity. (**A**) Overlap between differentially expressed genes from E2 vs. Control (*n* = 1699, yellow) and siARID1A vs. Control (*n* = 4128, green) in 12Z-ESR1 cells and genes with ARID1A promoter binding by ChIP-seq in 12Z cells (*n* = 3843, pink) as previously described [24]. (**B**) Violin plot of ChIP signal for ARID1A binding at 436 intersect genes (as in A) compared to all genes. The unpaired, 2-tailed Wilcoxon was used for analysis. (**C**) Expression log2 fold-change values of 436 intersect genes following treatment with E2, siARID1A, or both E2 and siARID1A. The paired, 2-tailed Wilcoxon test was used for analysis. Box-and-whiskers plotted in the style of Tukey without outliers. (**D**) Clustering of 436 genes based on relative gene expression in each condition. Clusters have been labeled as antagonistic (red for upregulted or blue for downregulated) or cooperative (purple) in their regulation by E2 and ARID1A. (**E**) ARID1A binding among intersect genes segregated into 4 groups based on their direction of gene expression change following E2 or siARID1A treatment. (**F**) Pathway enrichment analysis of 436 intersect genes for Gene Ontology (GO) Biological Process. (**G**) Pathway enrichment analysis of 436 intersect genes for MSigDB Hallmark pathways. (**H**) Left, fold-change values of 36 Hallmark EMT genes found among the 436 intersect genes following treatment with E2, siARID1A, or both E2 and siARID1A. The statistic represented is from the paired, 2-tailed Wilcoxon test. Box-and-whiskers plotted in the style of Tukey without outliers. Right, relative expression of individual 36 EMT intersect genes as a clustered heatmap. (**I**) Enrichment of DE genes affected by E2 treatment, ARID1A loss, or both for genes with active promoters directly inside of super-enhancer (SE, pink) (*n* = 163) (left), promoters within 50 kb of a super-enhancer (*n* = 493) (center), or genes linked to super-enhancer by the GeneHancer database (*n* = 1576) (right) compared to enrichment for all active genes (black). Hypergeometric enrichment was used for analysis.

We next considered the role of ARID1A binding in regulating estrogen-induced genes. We utilized our ARID1A ChIP-seq data from 12Z cells [24] and identified 436 genes with detected ARID1A promoter binding and differential gene expression following ARID1A loss or E2 treatment in 12Z-ESR1 cells (Figure 4A). As expected, the ARID1A binding at these gene promoters was higher than expected by chance (Figure 4B). Among these 436 genes, treatment with E2 generally decreased gene expression, while siARID1A treatment generally increased gene expression (Figure 4C). As such, most of these genes displayed a cooperative relationship in their regulation by ARID1A and E2 (Figure 4D). Intriguingly, ARID1A binding appeared highest at genes that downregulated following E2 treatment, regardless of the effect of differential gene expression following ARID1A loss (Figure 4E). Using these 436 genes, we performed gene set enrichment analysis for GO Biological Processes and MsigDB Hallmark pathways and identified cell adhesion and EMT as the top enriched pathways (Figure 4F,G). We identified 36 Hallmark EMT genes among the 436 intersect genes, and these genes also displayed mostly cooperative regulation by ARID1A and E2, suggesting that estrogen stimulation may lead to EMT downregulation or re-epithelialization (Figure 4H). Among these, we observed differential expression of *SERPINE1*, a gene regulated by ARID1A through repression of hyperacetylation of the *SERPINE1* super-enhancer [25]. *SERPINE1* expression is upregulated following ARID1A loss but repressed following E2 treatment (Figure 4H), suggesting ARID1A may normally be involved in its repression following estrogen stimulation. Furthermore, genes that were differentially expressed following E2 treatment, ARID1A knockdown, or both were enriched for genes regulated by active super-enhancers in 12Z cells (Figure 4I), as previously defined [25], which are known to regulate genes related to cell identity [39,40]. These results suggest that ARID1A and estrogen may govern epithelial cell identity in the endometrium by co-regulating cell identity genes governed by super-enhancers, where estrogen stimulation suppresses mesenchymal gene expression, assisted by ARID1A-SWI/SNF chromatin regulation, to promote epithelialization.

### 3.3. ARID1A Opposes PRC2-Mediated Repression of Estrogen-Mediated Progesterone Receptor Expression

**Figure 5:**
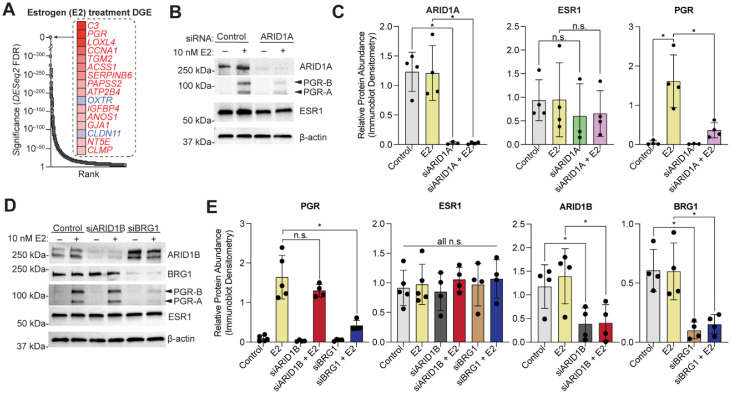
SWI/SNF mediates activation of PGR expression following E2 stimulation. (**A**) Rank visualization of top estrogen-regulated genes in 12Z-ESR1 cells. Red indicates upregulated genes, blue indicates downregulated genes. (**B**) Western blot analysis in 12Z-ESR1 cells following treatment with E2, siARID1A, or both E2 and siARID1A for ARID1A, PGR, and ESR1. PGR isoforms are indicated by arrowheads. β-Actin was used as a loading control. Blot is representative of 4 independent experiments. (**C**) Densitometry analysis of protein expression of ARID1A, PGR, and ESR1 following western blot as in panel B for control (silver), E2 (yellow), siARID1A (green) and siARID1A + E2 (pink). Densitometry values were normalized to β-Actin. Means ± SDs, *n* = 3–4. The paired *t*-test was used for analysis. (**D**) Western blot analysis in 12Z-ESR1 cells following treatment with E2, siBRG1, siARID1B, siBRG1+E2, or siARID1B+E2 for ARID1B, BRG1, PGR, and ESR1. PGR isoforms are indicated by arrowheads. β-Actin was used as a loading control. Blot is representative of 4 independent experiments. (**E**) Densitometry analysis of protein expression of ARID1B, BRG1, PGR, and ESR1 following western blot as in panel D for control (silver), E2 (yellow), siARID1B (charcoal), siARID1B + E2 (red), siBRG1 (brown) and siBRG1 + E2 (purple). Densitometry values were normalized to β-Actin. Means ± SDs, *n* = 4–5. The statistic is paired *t*-test. * *p* < 0.05; n.s. is non-significant.

We further investigated the role of ARID1A in the regulation of the progesterone receptor. Out of the 1699 genes empirically defined as estrogen-mediated in 12Z ESR1+ cells, PGR is one of the most highly induced estrogen target genes (Figure 5A), consistent with previous findings [7]. In EC patient samples, PGR expression is significantly correlated with ESR1 expression but is not correlated with the alternative estrogen receptor, ESR2 (Figure S2). However, following ARID1A loss in 12Z-ESR1 cells, *PGR* transcript induction by E2 is reduced (see Figure 1D). We confirmed the change in PGR protein expression by western blot, observing a ~75% reduction in PGR protein expression following ARID1A loss (Figure 5B,C). ARID1A loss had a similar effect on both PGR isoforms, the ~120 kDa PGR-B and the ~90 kDa PGR-A (Figure 5B). This result confirms that the decrease in *PGR* transcript following ARID1A loss translates to a decrease in PGR protein expression.

ARID1A is one of multiple SWI/SNF subunits implicated in human disease, as alternative SWI/SNF subunit configurations regulate chromatin in a tissue-dependent manner [62,63]. The catalytic subunit BRG1 is also mutated in EC and reduced expression has also been observed [64,65]. SWI/SNF complexes often contain both ARID1A and BRG1 [66], and these subunits co-regulate epithelial identity in the endometrial epithelium [27]. Therefore, we tested whether BRG1 loss also reduced estrogen-induced PGR expression. By using siRNA knockdown of BRG1 in 12Z-ESR1 cells, we observed a reduction in estrogen-induced PGR expression (Figure 5D,E). As an alternative to ARID1A, SWI/SNF complexes can incorporate the paralogous DNA-binding subunit, ARID1B [63], so we tested whether loss of ARID1B also affected PGR expression. Through knockdown of ARID1B by siRNA transfection into 12Z-ESR1 cells, we observed no change in estrogen-induced PGR expression (Figure 5D,E), suggesting a specific role for ARID1A-containing SWI/SNF complexes in regulating PGR upon estrogen stimulation in the endometrial epithelium.

**Figure 6:**
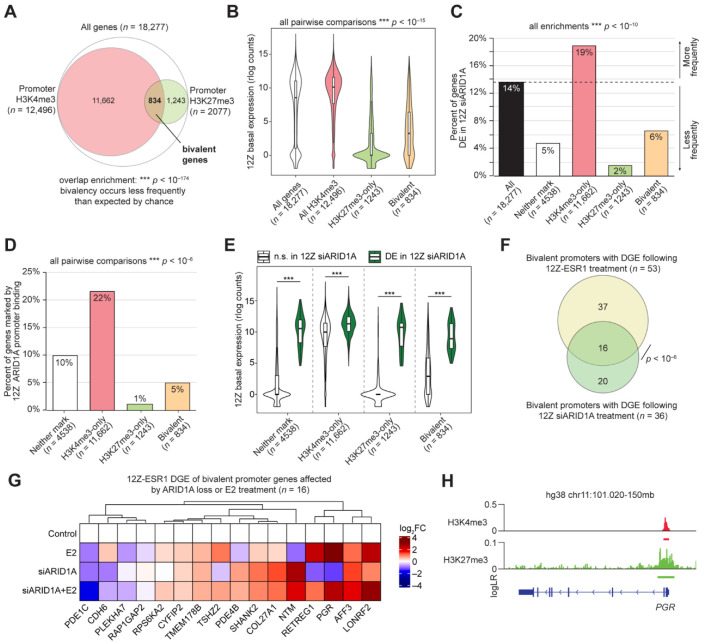
ARID1A regulation of bivalent gene promoters. (**A**) Proportional Euler diagram displaying overlap between genes with promoter H3K4me3 (*n* = 12,496, red) or H3K27me3 (*n* = 2077, green) among all expressed genes (*n* = 18,277, white). Hypergeometric enrichment was used for analysis. (**B**) Violin plot of 12Z basal expression of all genes with H3K4me3 at the promoter (red), genes with both H3K4me3 and H3K27me3 at the promoter (bivalent, yellow), and genes with only H3K27me3 at the promoter (green) relative to all genes (white). The unpaired, 2-tailed Wilcoxon test was used for analysis. 12Z gene expression from GSE121198 [24]. (**C**) Percent of genes with differential gene expression following siARID1A treatment of 12Z cells among genes with bivalent promoters (yellow), H3K4me3 only (red), H3K27me3 only (green), or neither (white) relative to all genes (black). Hypergeometric enrichment was used for analysis. (**D**) Percent of genes with ARID1A binding at the promoter among genes with bivalent promoters (yellow), H3K4me3 only (red), H3K27me3 only (green), or neither (white). The 2-tailed Fisher’s exact test was used for analysis. (**E**) Violin plot of basal expression of all genes with bivalent promoters, H3K4me4 only, H3K27me3 only, or neither, further segregated based on whether the gene exhibited differential gene expression following ARID1A loss (green) or not (white) in 12Z cells. The unpaired, 2-tailed Wilcoxon test was used for analysis. (**F**) Proportional Euler diagram displaying overlap between bivalent genes with differential gene expression in 12Z-ESR1 cells following E2 treatment (*n* = 53, yellow) and 12Z cells following ARID1A loss (*n* = 36, green) Hypergeometric enrichment was used for analysis (**G**) Differential expression heatmap of 16 genes from an overlapping group of panel F. (**H**) Genomic snapshot of ChIP signals for H3K4me3 (red) and H3K27me3 (green) at the PGR locus of 12Z cells. For signal tracks, the y-axis represents the assay signal-to-noise presented as the log-likelihood ratio (logLR) as reported by MACS2, and the small bars below the tracks represent replicate overlapping peaks. *** *p* < 0.001.

We next considered the regulation of *PGR* by ARID1A at the chromatin level. The SWI/SNF complex plays a role in developmental and cell identity processes through opposition to chromatin silencing by the Polycomb Repressive Complexes (PRCs) [67,68,69]. PRCs often play important roles in disease pathogenesis, and the catalytic subunit of PRC2, EZH2 (Enhancer of Zeste Homolog 2), is overexpressed in several cancers [70,71,72,73,74,75,76]. In normal cells, SWI/SNF actively removes PRC2 from chromatin via its remodeling activity [67,77,78,79]. Notably, inhibition of EZH2 has been suggested as a treatment for ARID1A-deficient ovarian clear cell carcinoma through a synthetic lethal relationship with ARID1A [80]. EZH2 acts specifically as a histone H3 lysine 27 (H3K27) methyltransferase, resulting in the H3K27 trimethyl (me3) histone modification at target chromatin [81]. The H3K27me3 mark is a repressive histone modification deposited by PRC2, while H3K4me3 is an activating chromatin modification that may prevent permanent silencing [82,83]. During development, chromatin can be modified with both H3K27me3 and H3K4me3, allowing for gene repression until activation is required [84,85]. Chromatin modified with both H3K27me3 and H3K4me3 is canonically referred to as bivalent chromatin [84,85]. We previously characterized the chromatin environment of 12Z cells through a survey of 5 histone modifications, including both H3K27me3 and H3K4me3, identifying an association between ARID1A and the activating modification H3K4me3 but not the repressive modification H3K27me3 [25]. Among promoters with either H3K4me3 or H3K27me3, bivalency occurred less frequently than expected by chance, with only ~7% of H3K4me3 promoters also marked with H3K27me3 (Figure 6A). As expected, gene promoters marked with H3K4me3 display high basal gene expression in 12Z cells, while promoter H3K27me3 is associated with low expression (Figure 6B). Bivalent gene promoters were associated with higher basal expression than H3K27me3 alone and lower expression than all H3K4me3 genes (Figure 6B). Genes marked with bivalent promoters were less likely than chance to be promoter-bound by ARID1A or differentially expressed upon ARID1A loss, though bivalent genes were more likely to be regulated by ARID1A than those marked by H3K27me3 alone (Figure 6C,D). Among genes with bivalent promoters, genes affected by ARID1A knockdown in 12Z cells have higher basal gene expression, although this is true for genes without bivalent promoters (Figure 6E). Overall, these results suggest that ARID1A is not associated with bivalent promoters globally. However, by comparing genes with bivalent promoters to our differential gene expression following E2 treatment or ARID1A loss, we observed 53 bivalent genes regulated by E2 and 36 bivalent genes affected by ARID1A loss (Figure 6F). These genes are summarized in Table 2. Among these, 16 genes with bivalent promoters were affected by both ARID1A loss and E2 treatment, including *PGR* (Figure 6G,H). These results suggest that a subset of genes regulated by both ARID1A and E2 may be antagonized by PCR2 upon ARID1A loss.

**Figure 7:**
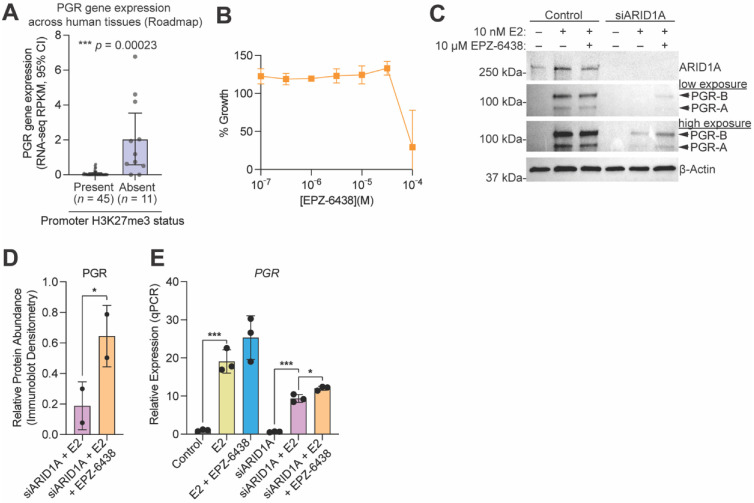
SWI/SNF antagonizes PCR2 to promote PGR expression following estrogen stimulation. (**A**) PGR gene expression (quantified by RPKM (Reads Per Kilobase of transcript, per Million mapped reads) at 95% confidence interval (CI)) in human tissue samples with (purple) vs. without (grey) promoter H3K27me3, taken from the NIH Epigenomics Roadmap. (**B**) Cell growth assay of 12Z cells treated with EPZ-6438 at concentrations from 100 nM to 100 μM. M is moles.(**C**) Western blot analysis in 12Z-ESR1 cells following treatment with E2, siARID1A or both, with or without 10 µM EPZ-6438 treatment, for ARID1A, PGR and ESR1. PGR isoforms are indicated by arrowheads. β-Actin was used as a loading control. Blot is representative of 2 independent experiments. (**D**) Densitometry analysis of protein expression of PGR following western blot as in panel C for siARID1A + E2 (pink) or siARID1A + E2 + EPZ-6428 (orange). Densitometry values were normalized to β-Actin are relative to the E2 only condition. Means ± SDs, *n* = 2. The statistic is paired *t*-test. (**E**) Quantitative PCR (qPCR) analysis of *PGR* gene expression 12Z-ESR1 cells following treatment with 10 nM E2 (yellow), 10 nM E2 + 10 µM EPZ-6438 (blue), siARID1A (green), siARID1A + E2 (pink), and siARID1A + 10 nM E2 + 10 µM EPZ-6438 (orange) relative to control (grey). Data represents 3 biological replicates, means ± SD. The unpaired *t*-test was used for analysis. * *p* < 0.05; *** *p* < 0.001.

**Figure 8:**
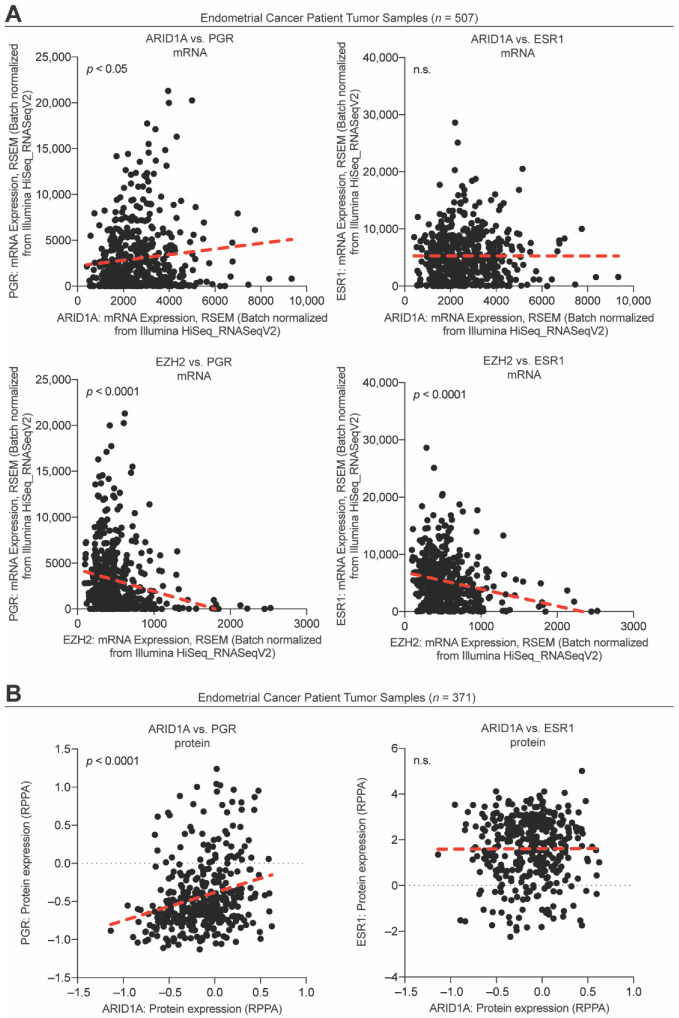
Correlations between PGR, ESR1, ARID1A, and EZH2 among endometrial cancer patient tumor samples. (**A**) Endometrial cancer tumor sample mRNA expression from 507 samples for ARID1A and PGR (upper left), ARID1A and ESR1 (upper right), EZH2 and PGR (lower left), and EZH2 and ESR1 (lower right). The red dashed line represents a linear regression. The statistical test used is the Pearson’s test. (**B**) Endometrial cancer tumor sample protein expression from 371 samples for ARID1A and PGR (left) and ARID1A and ESR1 (right). The red dashed line represents a linear regression. The statistical test used is the Pearson’s test. n.s. is non-significant.

**Figure 9:**
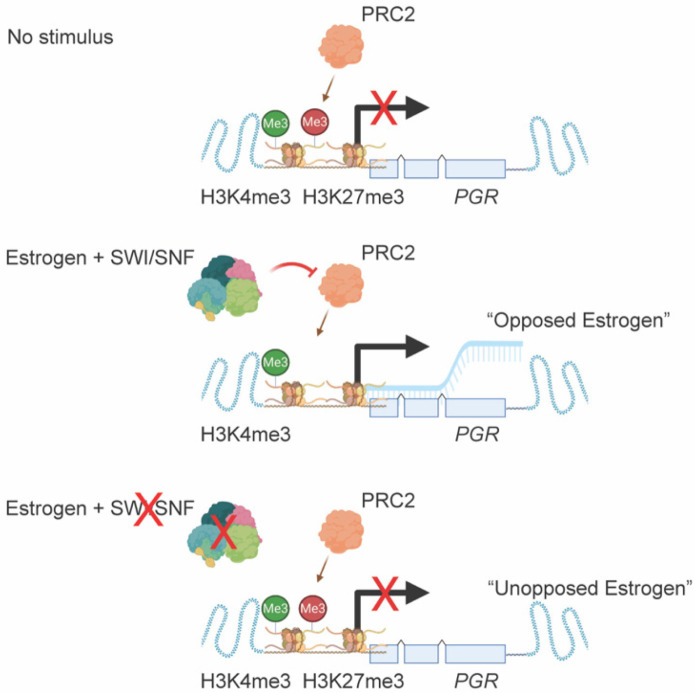
Model diagram representation of PGR (progesterone receptor) regulation by SWI/SNF (SWItch/Sucrose Non-Fermentable), PRC2 (polycomb repressive complex 2) and estrogen at sites with H3K27me3 (histone H3 lysine 27 trimethylation) and H3K4me3 (histone H3 lysine 4 trimethylation).

Following our observation that *PGR* is regulated by a bivalent promoter in 12Z cells, we utilized the NIH Epigenomics Roadmap data to investigate if bivalent chromatin may be a mechanism regulating *PGR* expression across human tissues. Among 56 tissue samples that had both H3K27me3 ChIP data and matched transcript data, we found that those samples with H3K27me3 at the *PGR* promoter had significantly lower *PGR* expression than those without promoter H3K27me3 (Figure 7A), further suggesting that *PGR* expression can be repressed by H3K27me3. As H3K27me3 is deposited by EZH2, we next tested whether *PGR* induction following E2 stimulation was being repressed by EZH2 enzymatic activity following ARID1A loss. To do so, we utilized a small molecule inhibitor of EZH2, EPZ-6438, which inhibits methyltransferase activity in a dose-dependent manner [86,87]. EPZ-6438 had no effect on 12Z cell growth at concentrations of 30 μM or below (Figure 7B). At a concentration of 10 μM, EPZ-6438 treatment of 12Z-ESR1 cells partially rescued the expression of both PGR protein and *PGR* mRNA in ARID1A knockdown cells treated with E2 (Figure 7C–E). These results are recapitulated in EC patient samples, as ARID1A expression is positively correlated with PGR expression (Figure 8A,B), while EZH2 expression is negatively correlated with both PGR and ESR1 expression (Figure 8A). These results support a role for EZH2 in repressing *PGR* in the context of ARID1A loss in the endometrial epithelium, leading to progesterone resistance and an “unopposed estrogen” phenotype in endometrial cancer (Figure 9).

## 4. Discussion

Here, we described roles for ARID1A and EZH2 in the regulation of PGR upon E2 stimulation. ARID1A co-regulates a large subset of genes regulated by estrogen, which may explain why ARID1A mutations are common in estrogen-dependent gynecologic diseases and cancers, wherein ARID1A mutations are observed in the epithelial compartment. Estrogen is a master regulator of the endometrial epithelium, and we found that both ARID1A and estrogen co-regulate epithelial identity genes, with ARID1A playing non-conventional roles in repression. ARID1A loss leads to EMT and invasion in the endometrial epithelium, and the results presented here suggest a role for wild-type ARID1A and estrogen signaling in the co-operative maintenance of epithelial identity. At other genes, ARID1A plays traditional activating roles following estrogen stimulation, which includes counteracting the repressive effects of PRC2 complexes at critical genes like *PGR*. Loss of either ARID1A or BRG1 SWI/SNF subunits is frequently observed in diseases in which PGR expression is lost, including breast cancer, in which ARID1A is mutated in 5% of cases [88] and ARID1A and BRG1 are known to play a role in response to ESR1 antagonists [89]. Positive correlations between ARID1A and PGR expression, and negative correlations between EZH2 and PGR/ESR1 expression, were also observed in breast cancer patient samples (Figure S3). Our results suggest a potential therapeutic application for EZH2 inhibitors in the restoration of progesterone sensitivity in these contexts.

The exact role of PGR in the epithelial component of the endometrium remains unclear. PGR is expressed in both the epithelial and stromal compartments of the endometrium. However, in vivo mouse studies and in vitro human cell line studies have demonstrated a role for stroma PGR, rather than epithelial PGR, in reducing epithelial proliferation following estrogen stimulation or apoptosis following ovariectomy [90,91,92,93,94]. Still, other mouse studies have demonstrated roles for epithelial PGR in regulating gene expression and reducing proliferation [95,96]. Epithelial PGR is reduced in progesterone-resistant ectopic endometriotic lesions [15], although it is unclear if the lesions showing PGR loss have impaired SWI/SNF activity. PGR expression correlates with the response of CAH and EC to progestin therapy, suggesting an important role for PGR in pathologies derived from the endometrial epithelium itself, as would be observed in endometrial cancer. 

Previous studies have shown that PGR expression is regulated through epigenetic mechanisms. The *PGR* promoter is rich in CpG islands [97]. DNA methylation leads to silencing of PGR-B in the eutopic endometrium of women with endometriosis [97,98], a phenomenon that may occur due to aberrant expression of DNA methyltransferases in endometriosis [99]. However, the regulation of PGR expression through histone methylation is relatively understudied. Expression of histone demethylase JARID1A can repress *PGR* expression through the removal of activating H3K4me3 marks [100,101]. PGR itself has been shown to upregulate EZH2 in the mammary epithelium [102]. EZH2 has been reported as a transcriptional activator of *ESR1* expression in breast cancer cells [103]. In the 12Z-ESR1 model, in which ESR1 was stably overexpressed, we did not explore the regulation of *ESR1* by EZH2, although EZH2 expression is negatively correlated with ESR1 expression in patient tumor tissues (Figure 8A,B). Endometriotic lesions have been characterized as hypermethylated at H3K4 relative to eutopic endometrium, and higher numbers of cells within lesions express nuclear H3K27me3 [104,105]. Our results show that endometrial pathologies harboring ARID1A mutations have altered steroid hormone signaling driven by EZH2 methyltransferase activity. This suggests a role for ARID1A and EZH2 in the regulation of PGR during normal endometrial cycling in response to estrogen and progesterone signaling, with differential expression being mediated by facultative chromatin modifications.

In EC, EZH2 expression is increased in tumor samples compared to normal tissue, and high EZH2 expression correlates with decreased progression-free survival or overall survival [106,107,108,109]. Increases in EZH2 expression have also been observed in CAH, but not simple hyperplasia [108]. Positive correlations have been demonstrated between EZH2 protein expression and stage, grade, and depth of invasion in endometrial cancer patients [110]. Knockdown of EZH2 in EC cell lines has been shown to reduce proliferation and invasion [108,110,111,112]. In the mouse uterus, epithelial EZH2 expression is induced by estrogen and inhibited by progesterone [113]. Knockout of both epithelial and stromal EZH2 in the mouse using a *Pgr-Cre* system [114] resulted in endometrial hyperplasia [113]. RNA-seq was performed on bulk uterine tissue from this model, and loss of EZH2 appeared to enhance estrogen-regulated pathways [115], although a later study found that only 7 genes were differentially expressed in a comparison of the wild-type epithelium and EZH2 knockout epithelium in this model [116]. Additional studies are needed to characterize the specific role of EZH2 in the endometrial epithelium and its relevance as a therapeutic target in EC. 

In the past decade, targeting of EZH2 in SWI/SNF-deficient cancers has been the focus of many studies. The synthetic lethal relationship was first identified in SMARCB1 deficient rhabdoid tumors and lymphomas, in which tumor phenotypes could be rescued by inhibition of EZH2 [67,86]. EPZ-6438 (also called tazemetostat) has been tested in a phase 1 study for refractory B-cell non-Hodgkin lymphoma and advanced solid tumors harboring somatic SWI/SNF mutations in which it was shown to have both antitumor activity and a favorable safety profile [117]. The therapeutic utility of EZH2 has been explored extensively in ovarian clear-cell cancer (OCCC), another gynecologic cancer with high rates of ARID1A mutation [118,119]. In epithelial OC, high expression of BRG1 expression is associated with improved survival, while high expression of EZH2 is predictive of poor outcome [120]. Loss of BRG1 in ARID1A-mutant OCCC cell line, TOV21G, promotes resistance to EZH2 inhibitors [121]. Endometriosis is a known risk factor for the endometroid and clear cell subtypes of OC [122,123], and the peritoneal fluid from women with endometriosis has been shown to induce expression of EZH2 and H3K27me3 in OC cell lines [124]. EZH2 has also been shown to mediate mesenchymal-to-epithelial transition of OC-associated stromal stem cells, which promote metastasis [125]. EZH2 inhibition restores the expression of *ESR1* in high-grade serous OC cells [126]. Histone deacetylase (HDAC) inhibitors and EZH2 inhibitors have been shown to synergize in BRG1-deficient small cell carcinoma of the ovary, hypercalcemic type cells through increased H3K27 acetylation [127]. In the present study, we utilized sub-lethal concentrations of EPZ-6438 and showed an effect on PGR expression, suggesting that EZH2 inhibition may restore progesterone sensitivity in EC and other progesterone-resistant, SWI/SNF deficient gynecologic pathologies, thus blocking the growth-promoting effects of estrogen.

## Figures and Tables

**Table 1:** Gene Set Enrichment Analysis for MsigDB Hallmark Pathways. Positive values indicate pathway upregulation in the respective treatment condition, and negative values indicate pathway downregulation. * *p* < 0.05.

	Normalized Enrichment Score (NES)		
Hallmark Pathway	E2 vs. Control	siARID1A + E2 vs. siARID1A	siARID1A + E2 vs. E2
Adipogenesis	1.73 *	1.77 *	–1.50 *
Allograft Rejection	1.00 *	–1.30*	1.29 *
Androgen Response	1.56 *	1.65 *	–1.46 *
Angiogenesis	1.70 *	1.65 *	1.23
Apical Junction	1.37 *	–1.17	1.34 *
Apical Surface	1.45 *	–1.13	1.42 *
Apoptosis	1.55 *	1.38 *	1.40 *
Bile Acid Metabolism	1.52 *	1.16	–1.33
Cholesterol Homeostasis	1.34 *	1.23	–1.20
Coagulation	1.41 *	1.61 *	1.54 *
Complement	1.59 *	1.62 *	1.54 *
DNA Repair	–1.05	–1.36 *	–1.14
E2F Targets	–1.25	–1.28	0.86
Epithelial Mesenchymal Transition	1.38 *	–1.36 *	1.54 *
Estrogen Response Early	1.52 *	1.52 *	–1.40 *
Estrogen Response Late	1.59 *	1.54 *	–1.47 *
Fatty Acid Metabolism	2.05 *	1.67 *	–1.45 *
G2M Checkpoint	–0.70	1.06	1.15
Glycolysis	1.57 *	1.62 *	1.35 *
Hedgehog Signaling	–0.99	–1.23 *	1.35 *
Heme Metabolism	1.70 *	1.55 *	–1.24
Hypoxia	1.60 *	1.57 *	1.30
Il2 Stat5 Signaling	1.64 *	1.62 *	1.47 *
Il6 Jak Stat3 Signaling	1.22	–1.44 *	1.63 *
Inflammatory Response	1.23 *	–1.23	1.52 *
Interferon Alpha Response	1.32	–1.12	1.43
Interferon Gamma Response	1.29	1.27 *	1.38 *
Kras Signaling Dn	1.29 *	1.19	–1.32
Kras Signaling Up	1.82 *	1.33 *	1.36 *
Mitotic Spindle	0.95	1.01	1.19
MTORC1 Signaling	1.63 *	1.26	–1.30
Myc Targets V1	–1.08	–1.32 *	0.94
Myc Targets V2	–1.25 *	–1.41 *	1.19 *
Myogenesis	1.26	1.41 *	–1.16
Notch Signaling	0.99	1.05	1.32 *
Oxidative Phosphorylation	0.98	1.23	–1.25
P53 Pathway	1.85 *	1.90*	1.43 *
Pancreas Beta Cells	1.47	1.51*	1.20
Peroxisome	1.74 *	1.65*	–1.33 *
PI3K Akt MTOR Signaling	1.33	1.36*	–1.58 *
Protein Secretion	1.24	–0.67	–1.40 *
Oxygen Species Pathway	0.87	–1.11	–1.08
Spermatogenesis	1.47 *	1.53 *	–1.19
TGF-Beta Signaling	1.84 *	1.86 *	1.51 *
TNF-Alpha Signaling Via NFkB	1.70 *	1.51 *	1.46 *
Unfolded Protein Response	1.75 *	0.90	1.45 *
UV Response Dn	1.66 *	–1.23 *	1.51 *
UV Response Up	1.41 *	1.65 *	1.42 *
Wnt Beta Catenin Signaling	–1.01	1.17	1.03
Xenobiotic Metabolism	1.67 *	1.73 *	–1.50 *

**Table 2:** Genes with bivalent promoters affected by E2 treatment or ARID1A knockdown.

**Genes with Bivalent Promoters Differentially Expressed Upon E2 Treatment of 12Z-ESR1 Cells (*n* = 53)**					
AFF3	CYFIP2	LDHB	PDE4B	RAP1GAP2	SVEP1
APCDD1	ERG	LONRF2	PDE4D	RETREG1	SYBU
ASIC2	ESR1	LPIN1	PDGFD	RNF152	TGFA
BMP6	GFRA2	MAST4	PGR	RPS6KA2	TMEM178B
C4orf19	GUCY1A2	MKX	PLCB1	SHANK2	TMOD1
C4orf3	HAND1	NPY1R	PLEKHA7	SLC47A1	TPD52L1
CACNB4	ISM1	NRXN3	PRKAG2	SLCO5A1	TSHZ2
CDH6	ISOC1	NTM	PRR15	SNCAIP	WNT16
COL27A1	KIAA1217	PDE1C	PTGER4	SOX5	
**Genes with** **Bivalent Promoters Differentially Expressed Upon ARID1A Knockdown in 12Z Cells (*n* = 36)**					
AFF3	COL27A1	FAM131B	MOCS1	PLAGL1	RPS6KA2
ANK3	CYFIP2	FMN1	NR3C2	PLEKHA7	SHANK2
C17orf51	DEPDC1B	FOXQ1	NTM	RAP1GAP2	STOX2
CDH6	DLGAP4	GJB2	PDE1C	RETREG1	TMEM178B
CELF2	EIF4E3	LONRF2	PDE4B	RNF220	TSHZ2
CHRM3	ELOVL7	MAD2L2	PGR	RPL38	VDR
**Overlapping Genes (*n* = 16)**					
AFF3	CYFIP2	PDE1C	PLEKHA7	RPS6KA2	TSHZ2
CDH6	LONRF2	PDE4B	RAP1GAP2	SHANK2	
COL27A1	NTM	PGR	RETREG1	TMEM178B	

## Data Availability

All new data generated in this study have been deposited to GEO at accession GSE195503. Previously generated and re-analyzed data were retrieved from GEO at accessions GSE121198 and GSE148474.

## Supplementary Materials

The following supporting information can be downloaded at: https://www.mdpi.com/article/10.3390/cells11061000/s1, Figure S1: Expression of ESR1 among ARID1A wild-type and ARID1A mutant endometrial cancer cell lines; Figure S2: Correlations between steroid hormone receptors among endometrial cancer patient tumor samples; Figure S3: Correlations between PGR, ESR1, ARID1A and EZH2 among breast cancer patient tumor samples, Figure S4: Uncropped western blots.
