# Supplementary material for: SWI/SNF Antagonism of PRC2 Mediates Estrogen-Induced Progesterone Receptor Expression
Source: Cells. 2022 Mar 15;11(6):1000. doi: 10.3390/cells11061000 (PMC8946988; doi:10.3390/cells11061000)
Supplement: Supplementary file 1 [file cells-11-01000-s001.zip › cells-1603686-supplementary.pdf]

cells

MDPI

Supplementary Material

SWI/SNF Antagonism of PRC2 Mediates Estrogen-Induced Progesterone Receptor Expression

Mike R. Wilson, Jake J. Reske, Julie Koeman, Marie Adams, Niraj R. Joshi, Asgerally T. Fazleabas and Ronald L. Chandler

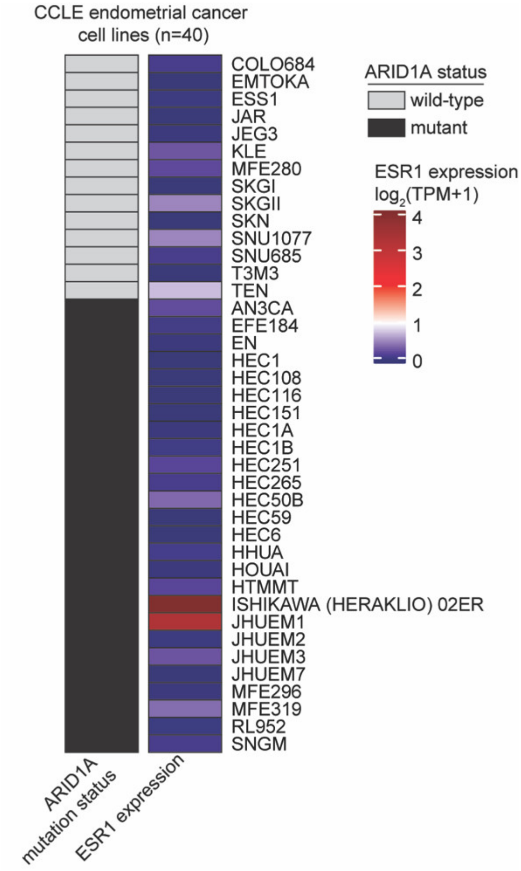

Figure S1. Expression of ESR1 among ARID1A wild-type and ARID1A mutant endometrial cancer cell lines. ARID1A mutation data and ESR1 mRNA expression data was taken from the Cancer Cell Line Encyclopedia (CCLE). Cell lines are listed alphabetically, segregated by ARID1A mutation status.

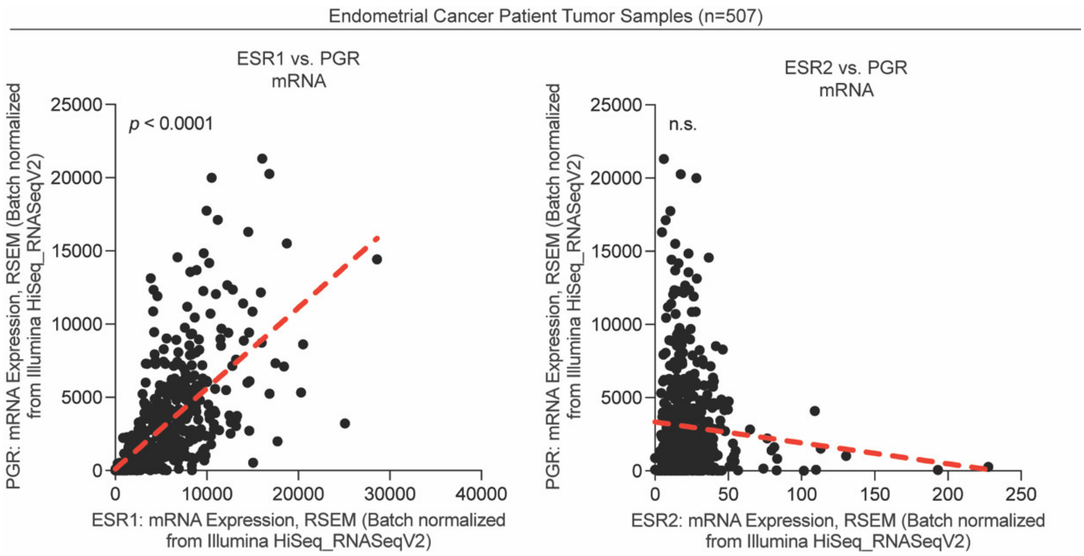

Figure S2. Correlations between steroid hormone receptors among endometrial cancer patient tumor samples. Endometrial cancer tumor sample mRNA expression for ESR1 and PGR (left) or ESR2 and PGR (right). Red dashed line represents linear regression. Statistic is Pearson's.

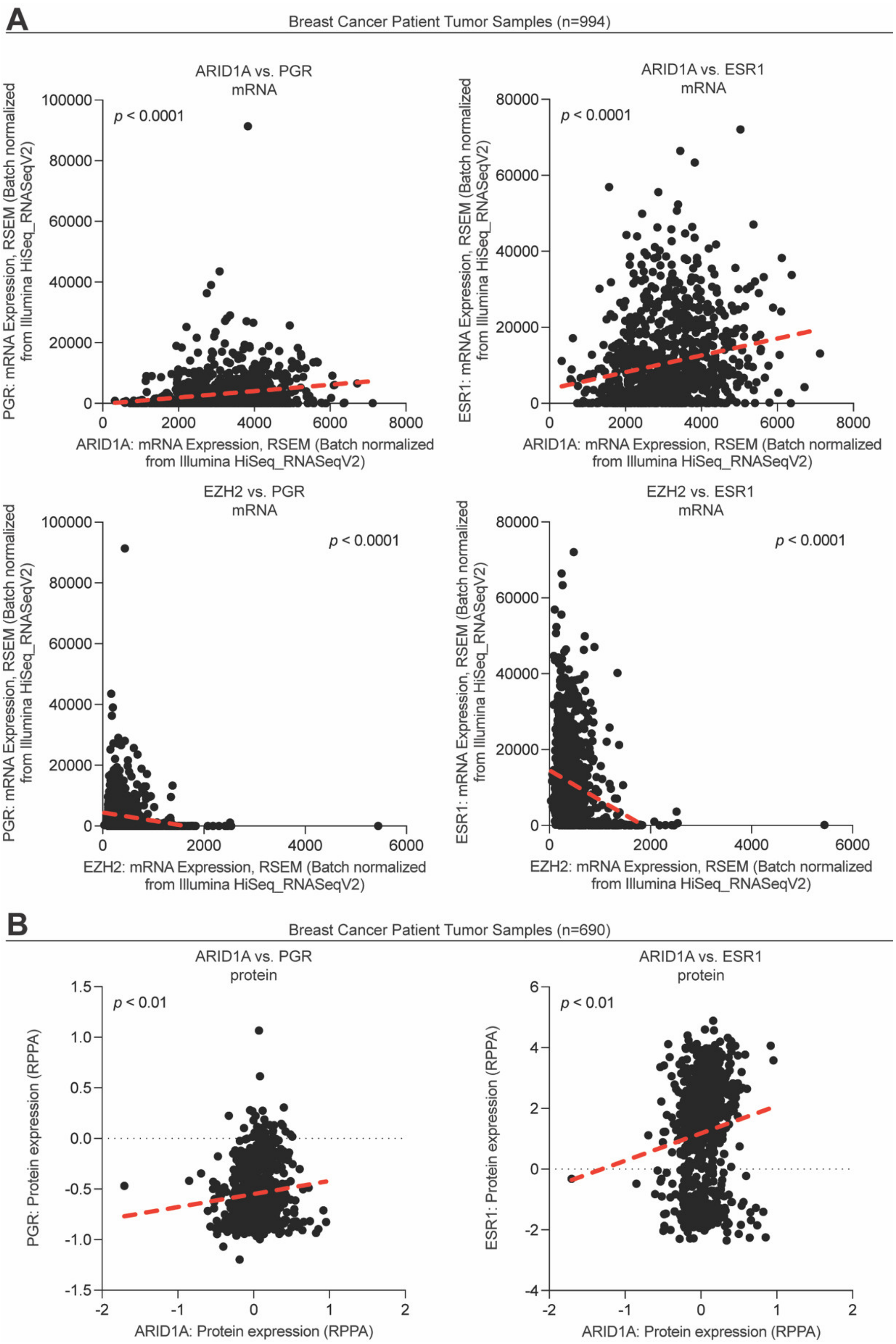

Figure S3. Correlations between PGR, ESR1, ARID1A and EZH2 among breast cancer patient tumor samples. (**A**) Breast cancer tumor sample mRNA expression from 994 samples for ARID1A and PGR (upper left), ARID1A and ESR1 (upper right), EZH2 and PGR (lower left) and EZH2 and ESR1 (lower right). Red dashed line represents linear regression. Statistic is Pearson's. (**B**) Breast cancer tumor sample protein expression from 690 samples for ARID1A and PGR (left), ARID1A and ESR1 (right). Red dashed line represents linear regression. Statistic is Pearson's.

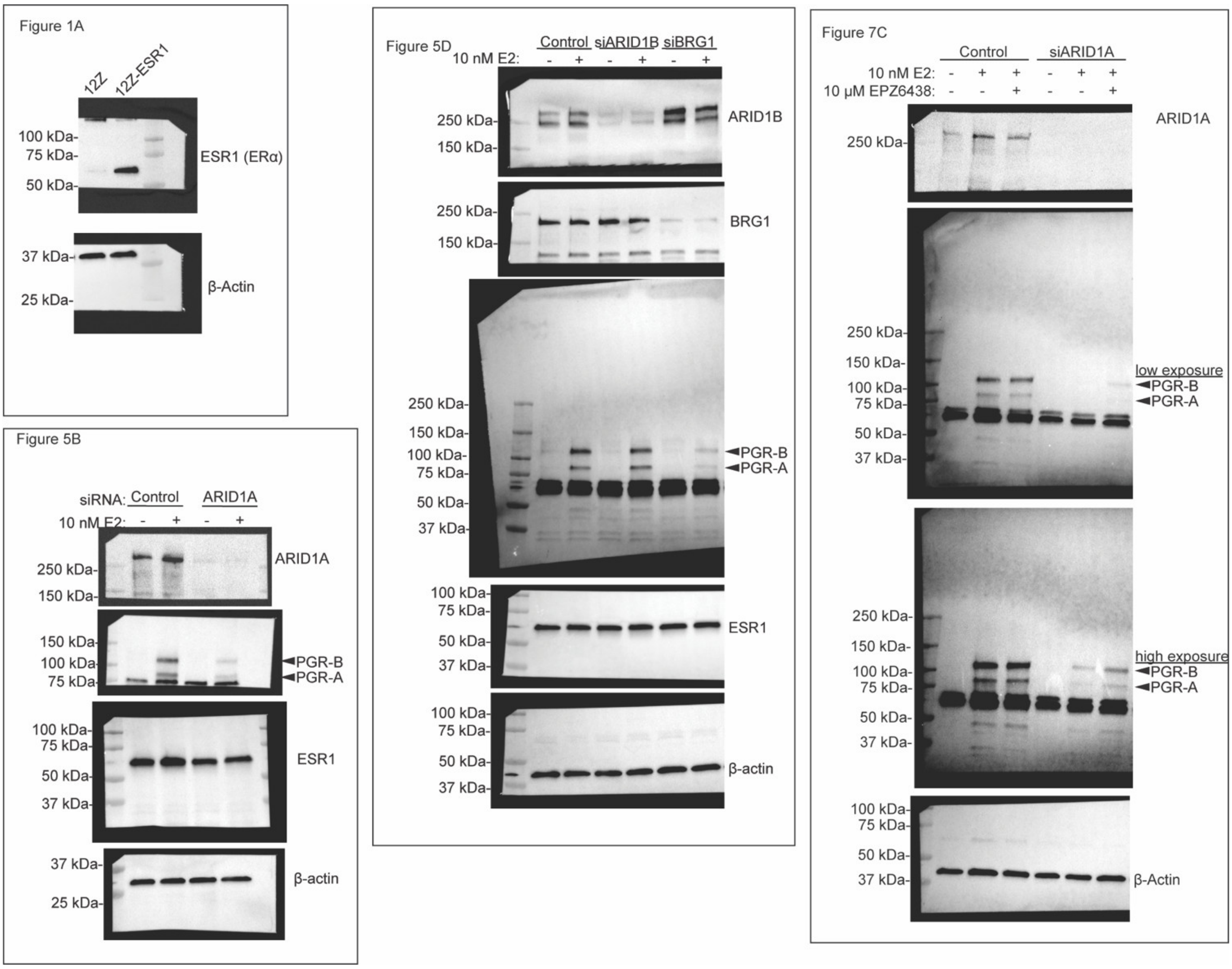

Figure S4. Uncropped western blots.
